# Multicenter longitudinal cross-sectional study comparing effectiveness of serratus anterior plane, paravertebral and thoracic epidural for the analgesia of multiple rib fractures

**DOI:** 10.1136/rapm-2019-101119

**Published:** 2020-03-11

**Authors:** Laura Beard, Carl Hillermann, Emma Beard, Sue Millerchip, Rajneesh Sachdeva, Fang Gao Smith, Tonny Veenith

**Affiliations:** 1 Department of Anaesthesia and Critical Care, Queen Elizabeth Hospital, Birmingham, UK; 2 Department of Anaesthesia, University Hospitals Coventry and Warwickshire, Coventry, UK; 3 Research Department of Behavioural Science and Health, UCL, University College London, London, UK; 4 Birmingham Acute Care Research Group, University of Birmingham, Birmingham, UK

**Keywords:** acute pain, truncal blocks, regional anesthesia

## Abstract

**Background:**

There is a paucity of data comparing effectiveness of various techniques for pain management of traumatic rib fractures. This study compared the quality of analgesia provided by serratus anterior plane (SAP) catheters against thoracic epidural (TEA) or paravertebral catheters (PA) in patients with multiple traumatic rib fractures (MRFs).

**Methods:**

354 patients who received either SAP, TEA or PA at two tertiary referral major trauma centers in the UK were included (2016–2018). Primary outcome were change in inspiratory volumes and pain scores. Secondary outcomes included in-hospital mortality, along with the length of stay in hospital and critical care. Data were analyzed using linear, log-binomial and negative binomial regression models.

**Main results:**

Across all blocks, there was a mean (SD) increase in inspiratory volume postblock of 789.4 mL (479.7). Ninety-eight per cent of all participants reported moderate/severe pain prior to regional analgesia, which was reduced to 34% postblock. There was no significant difference in the change in inspiratory volume or pain scores between the TEA, PA or SAP groups. Overall crude mortality was 13.2% (95% CI 7.8% to 18.7%). In an adjusted analysis and compared with TEA, in-hospital mortality was similar between groups (relative risk (RR) 0.4, 95% CI 0.1 to 1.0) and (RR 0.5, 95% CI 0.2 to 1.6) for SAP and PA, respectively.

**Conclusion:**

SAP, TEA and PA all appear to offer the ability to reduce pain scores and improve respiratory function.

## Introduction

Patients with thoracic trauma are at significant risk of morbidity and mortality from their initial thoracic injury or as a consequence of secondary insults such as atelectasis, pneumonia and acute respiratory distress syndrome. Pneumonia, as a complication of multiple rib fractures (MRFs), is diagnosed in 11%–31% of patients and associated with increased mortality.^1–3^ Effective analgesia has been shown to reduce the risk of respiratory complications by enabling the patient to deep breath, cough and facilitate early mobilization.^4–6^

Regional analgesic techniques such as thoracic epidural (TEA) and paravertebral catheters (PA) have long been used as the ‘gold standard’ method in the multimodal approach of pain management. Their use, however, is limited due to concomitant injuries (such as spinal and head injuries), contraindications (such as trauma-related coagulopathy and anticoagulant medications), multimorbidity (eg, aging trauma population) and inability to position the patient during the acute phase. Current literature reports TEA use between 9.9% and 18.4% in this group, with opioid analgesia, dominant.^1 2 7 8^

Thoracic wall fascial plane blocks with their ease of insertion, absence of beta-blockade effect with hemodynamic stability and safety are a welcome addition to the analgesic armamentarium in patients with MRFs.^9^ Many fascial plane techniques have been described, they include the serratus anterior plane (SAP), erector spinae plane (ESP) and rhomboid intercostal blocks, although high-quality data on their efficacy is lacking.^10^ Some blocks, such as the ESP, paravertebral and thoracic epidural, require patient cooperation in a lateral or sitting position during block insertion. This is often impossible in patients with significant thoracic, head or pelvic injuries after polytrauma. SAP blocks can be performed in the supine position with minimal repositioning. The evidence base for SAP analgesia in those with MRFs is limited to mainly case reports and case series.

Pain scores can be static (when the patient is at rest) or dynamic (on movement and coughing). Dynamic pain scores are more pertinent in patients with MRFs as this would determine their ability to breathe deeply, cough and mobilize when they are most vulnerable to develop respiratory complications^1 11^ For these reasons, we believe that a functional physiological assessment of analgesic adequacy assessed by measuring inspiratory volumes (incentive spirometry) should be a mandated outcome measure along with standard pain scores. This combined approach provides a better reflection of analgesic adequacy in this cohort.

The primary aim of this study was to compare the quality of analgesia, assessed by dynamic pain scores and inspiratory volumes, between SAP, TEA and PA catheters in a group of patients with traumatic MRFs. The secondary aim was to investigate if there was a difference in patient outcome between the three groups by analyzing the length of stay (LOS) and mortality data. Although systematic reviews have analyzed studies comparing TEA, PA, intercostal nerve blocks and opiate based treatments, to our knowledge, this is the first study to compare the effectiveness of SAP to PA or TEA.^12 13^

## Methods

### Design

We included all consecutive adults with MRFs who received SAP, TEA or PA catheters and were admitted to hospital between 2016 and 2018 in two tertiary referral major trauma centers in the UK. Patients who had more than one type of regional anesthesia or who died within 24 hours of admission to hospital were excluded. Outcome data were collected from existing pain databases and patient records. This was merged with data from a high-quality national database provided by the Trauma Audit Research Network.

### Measures

#### Primary outcome

The primary outcome measure was block effectiveness defined by a change in inspiratory volumes (mL) and dynamic pain scores, recorded before and after block insertion (within 90 min). Inspiratory volumes were measured with an incentive spirometer (mL). Pain scores were measured by a 4-point Likert Verbal Rating Scale (VRS) using standardized categories.^14^ Pain scores of 0–3 were recorded, where 0=no pain, 1=mild pain, 2=moderate pain and 3=severe pain. The four-point pain score was selected as this was the adopted practice in participating sites. Previous studies have shown that responses to the VRS yield similar precision and reliability as visual analog scales (VAS) and are often viewed as simpler to complete.^15 16^

#### Secondary outcomes

Secondary outcome measures included the overall LOS in hospital, LOS in critical care and in-hospital mortality.

#### Covariates

Covariates included demographic data on age, sex, comorbid state as assessed by Charlson Comorbidity Index (CCI), injury severity score (ISS), abbreviated injury score (AIS) for each body system and outcome (dead/alive). Data on mortality are provided from in-hospital patient deaths before discharge. Rib fracture score (RFS) was captured from the electronic patient records; if not available, the investigators calculated it from the trauma CT scan (RFS=(number of breaks × side factor)+age factor).^6^ Surgical rib fixation and intubation status at the time of regional anesthesia was also documented.

### Statistical analysis

Data were analyzed in R studio. Alpha was set to 0.05. All tests were two sided; thus, the null hypothesis reflected a belief that associations could be in either direction. Given that this is an exploratory analysis, we did not adjust for multiple comparisons. Differences in participant characteristics were assessed with analysis of variance (ANOVA) and χ^2^ tests for categorical variables. Post hoc analyses were conducted for any significant omnibus results using the Tukey Honest Significant Differences for ANOVA and standardized residuals for χ^2^.

**Figure 1:**
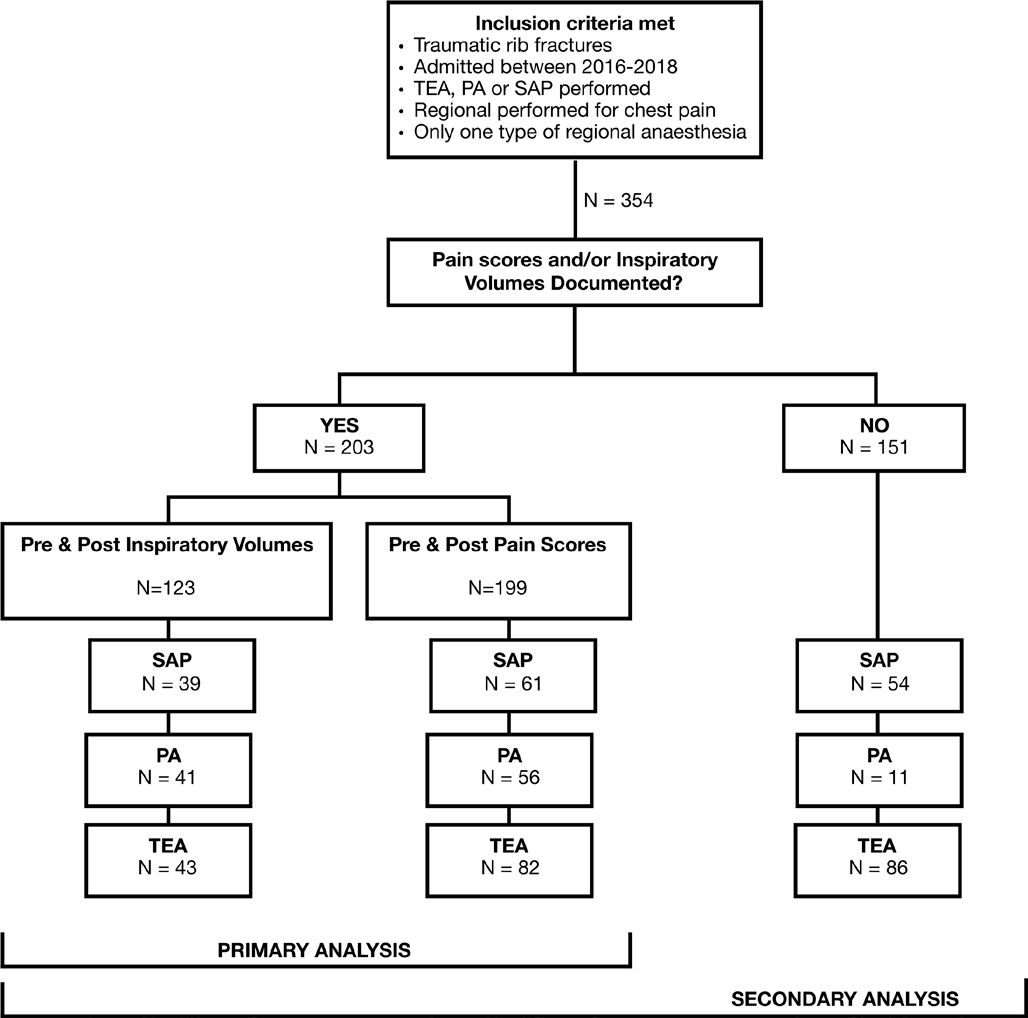
Inclusion criteria for each stage of the analysis. PA, paravertebral catheter; SAP, serratus anterior plane; TEA, thoracic epidural.

Missing data were handled using a pairwise deletion approach. Missing data were as follows for the sample overall who had either a PA, TEA or SAP catheter (n=354): age: n=3, ISS: n=8, RFS: n=31, CCI score: n=2, sex: n=3, isolated or polytrauma: n=3, mechanism: n=20, mechanism type: n=20, most severely injured body region: n=20, AIS score for the head, face, thorax, abdomen, spine, pelvis, limbs and other: n=20, preinspiratory and postinspiratory volume: n=231 and pre and post pain scores: n=155. Missing pain scores and inspiratory volumes were a result of patient inability to perform the tests due to intubation (n=50), confusion/reduced conscious level (n=8) and patient refusal (n=2). The remaining missing data had no reason provided. Figure 1 shows the inclusion criteria for each stage of the analysis.

Generalized linear models specifying the binomial family and log link function (log binomial regression) were used to assess difference in the number of participants moving from reporting severe/moderate pain preblock to mild/no pain postblock and in-hospital mortality. Generalized linear models specifying the Gaussian family and identify link function (similar to linear regression) were used to assess differences in change in inspiratory volume from the preblock to postblock measurement. Finally, generalized linear models specifying the negative binomial family with log link function (negative binomial regression) were used to assess differences in LOS in hospital/critical care among the three groups. Negative binomial models were used instead of Poisson regression due to the presence of overdispersion. Poisson and negative binomial models are recommended when predicting LOS as data are often skewed.^17–19^

Unadjusted and adjusted models are reported for each outcome. We adjusted for confounders that were chosen a priori, based on their expected associations with MRFs. For pain and inspiratory volumes, the following covariates were adjusted for: age, gender, ISS and RFS score. For in-hospital mortality and LOS, the following covariates were adjusted for: age, gender, ISS, RFS score, CCI, most severely injured body region (head, chest and other), surgical rib fixation and isolated chest injury versus polytrauma.

## Results

Data were collected on 354 patients who had either SAP (n=117), PA (n=68) or TEA (n=169). table 1 shows the characteristics of participants overall and as a function of the block type they received. Differences were assessed using χ^2^ tests and ANOVAs.

**Table 1:** Characteristics of participants overall and as a function of block type

	Overall	SAP	PA	TEA	P value*
Age, mean (SD)	61.3 (18.4)	70.0 (18.4)	65.5 (17.0)	59.9 (18.8)	0.50
ISS score, mean (SD)	23.9 (11.2)	26.9 (12.7)	18.2 (8.2)	24.1 (10.2)	0.10
RFS, mean (SD)	15.5 (9.5)	15.1^a^ (9.1)	11.1^b^ (4.8)	17.6^a^ (10.6)	0.02
CCI, mean (SD)	2.6 (2.3)	2.5 (2.4)	3.1 (2.3)	2.5 (2.2)	0.95
AIS scores, mean (SD)					
Head	0.8 (1.6)	1.2^a^ (1.9)	0.4^b^ (1.0)	0.7^b^ (1.5)	0.02
Face	0.2 (0.6)	0.4 (0.8)	0.1 (0.4)	0.2 (0.6)	0.06
Thorax	3.8 (0.6)	3.7^a^ (0.7)	3.6^a^ (0.7)	3.96^b^ (0.6)	0.01
Abdomen	0.6 (1.2)	0.7 (1.3)	0.5 (1.1)	0.6 (1.1)	0.58
Spine	0.8 (1.2)	1.1 (1.2)	0.5 (0.9)	0.9 (1.1)	0.23
Pelvis	0.5 (1.2)	0.6 (1.4)	0.3 (1.00.)	0.5 (1.2)	0.62
Limbs	1.0 (1.1)	1.2 (1.2)	0.5 (0.9)	1.2 (1.1)	0.80
Other	0.1 (0.3)	0.1^a^ (0.4)	<0.1^b^ (0.2)	<0.1^a^ (0.2)	0.02

					P value†
Gender, n (%)					
Female	98 (27.9)	27 (23.5)	29 (42.65)	42 (25.00)	
Male	253 (72.1)	88 (76.5^a^)	39 (57.35^b^)	126 (75.00^a^)	0.01
Trauma type, n (%)					
Polytrauma	258 (73.5)	90 (78.3)	38 (55.88)	130 (77.38)	
Isolated	93 (26.5)	25 (21.7^a^)	30 (44.1^b^)	38 (22.6^a^)	<0.01
Surgical rib fixation, n (%)					
Yes	74 (22.)	22 (19.6)	14 (23.3)	38 (25.0)	
No	250 (77.2)	90 (80.4)	46 (76.7)	114 (75.0)	0.59
Intubated at time of block, n (%)					
Yes	50 (24.8)	24 (28.9)	1 (4.0)	25 (26.6)	
No	152 (75.3)	59 (71.1^a^)	24 (96.0^b^)	69 (73.4^a^)	0.04
Mechanism, n (%)					
Fall <2 m	107 (32.)	35 (32.1^a^)	32 (48.5^b^)	40 (25.2^b^)	
Fall >2 m	55 (16.5)	16 (14.7)	11 (16.7)	28 (17.6)	
Vehicle	148 (44.)	53 (48.6^a^)	18 (27.3^b^)	77 (48.4^b^)	
Other	24 (7.2)	5 (4.6)	5 (7.6)	14 (8.8)	0.02
Most severely injured region, n (%)		
Chest	247 (4.0)	64 (58.7^a^)	57 (86.4^b^)	126 (79.3^b^)	
Head	26 (7.8)	15 (13.8^a^)	2 (3.0^b^)	9 (5.7^b^)	
Other	61 (18.3)	30 (27.5^a^)	7 (10.6^b^)	24 (15.1^b^)	<0.001

If letters differ ^(a/b)^, they indicate significant differences in the post hoc comparisons at alpha 0.05.

*P value from ANOVA.

†P value from χ^2^ test.

AIS, abbreviated injury score; ANOVA, analysis of variance; CCI, Charlson Comorbidity Index; ISS, injury severity score; PA, paravertebral catheter; RFS, rib fracture score; SAP, serratus anterior plane; TEA, thoracic epidural.

The PA group had a significantly lower proportion of patients with multiple injuries (polytrauma), endotracheal intubation and significant head injuries. The PA group also had significantly lower rib fracture (RFS) scores representing less severe chest trauma. The patient characteristics in the SAP and TEA groups were more comparable. However, the SAP group did have significantly more patients with severe head injuries compared with TEA and PA. The adjusted models selected aimed to account for these differences.

### Inspiratory volumes

**Figure 2:**
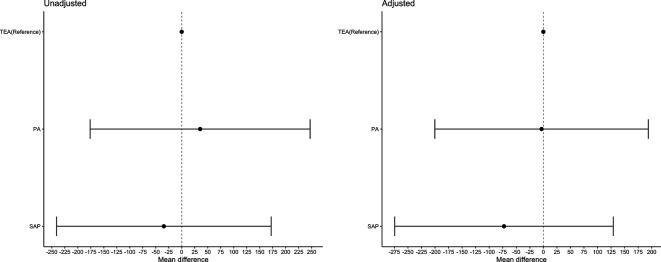
Mean difference (CIs) in inspiratory volume changes between block types for the unadjusted and adjusted linear regression models. Adjusted covariates: age, gender, ISS and RFS score. ISS, injury severity score; RFS, rib fracture score.

All groups saw a clinically significant mean increase in inspiratory volume over time of 789.4 mL (SD 479.7 mL). The mean increase for each block type individually was: SAP 825.6 mL (SD 519.8 mL), PA 790.2 mL (SD 515.4 mL) and TEA 755.8 mL (SD 410.6 mL). There was no statistically significant difference in the magnitude of this change between the TEA and the PA or SAP groups (see figure 2).

### Pain scores

**Figure 3:**
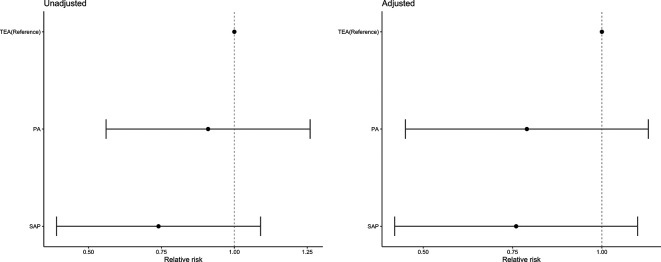
Relative risks (CIs) for the unadjusted and adjusted log binomial models comparing the number of participants reporting a change in their pain scores from severe/ moderate to mild/none. Adjusted covariates: age, gender, ISS and RFS score. ISS, injury severity score; RFS, rib fracture score.

Table 2 shows the pre and post pain scores overall and as a function of block type. Across all blocks, approximately 98% of all participants reported moderate/severe pain prior to regional analgesia, which was reduced to 34% following block placement. Of those who had moderate/severe pain in the preblock period, 55.17% (n=32) in the SAP group, 50.00% (n=28) in the PA group and 40.74% (n=33) in the TEA group reported no pain/mild pain postblock. The probability of changing from higher to lower levels of pain did not differ between groups (figure 3).

**Table 2:** Prepain and postpain scores overall and as a function of block type

	Preblock	Postblock
Overall, n (%)		
No pain	1 (0.5)	20 (10.1)
Mild pain	3 (1.5)	111 (55.8)
Moderate pain	42 (21.1)	66 (33.2)
Severe pain	153 (76.9)	2 (1.0)
SAP, n (%)		
No pain	1 (1.6)	7 (11.5)
Mild pain	2 (3.3)	27 (44.3)
Moderate pain	10 (16.4)	25 (41.0)
Severe pain	48 (78.7)	2 (3.3)
PA, n (%)		
No pain	0 (0.0)	2 (3.6)
Mild pain	0 (0.0)	36 (64.3)
Moderate pain	13 (23.2)	18 (32.1)
Severe pain	43 (76.8)	0 (0.0)
TEA, n (%)		
No pain	0 (0.0)	11 (13.4)
Mild pain	1 (1.2)	48 (58.5)
Moderate pain	19 (23.8)	23 (28.1)
Severe pain	62 (75.6)	0 (0.0)

PA, paravertebral catheter; SAP, serratus anterior plane; TEA, thoracic epidural.

### Mortality

**Figure 4:**
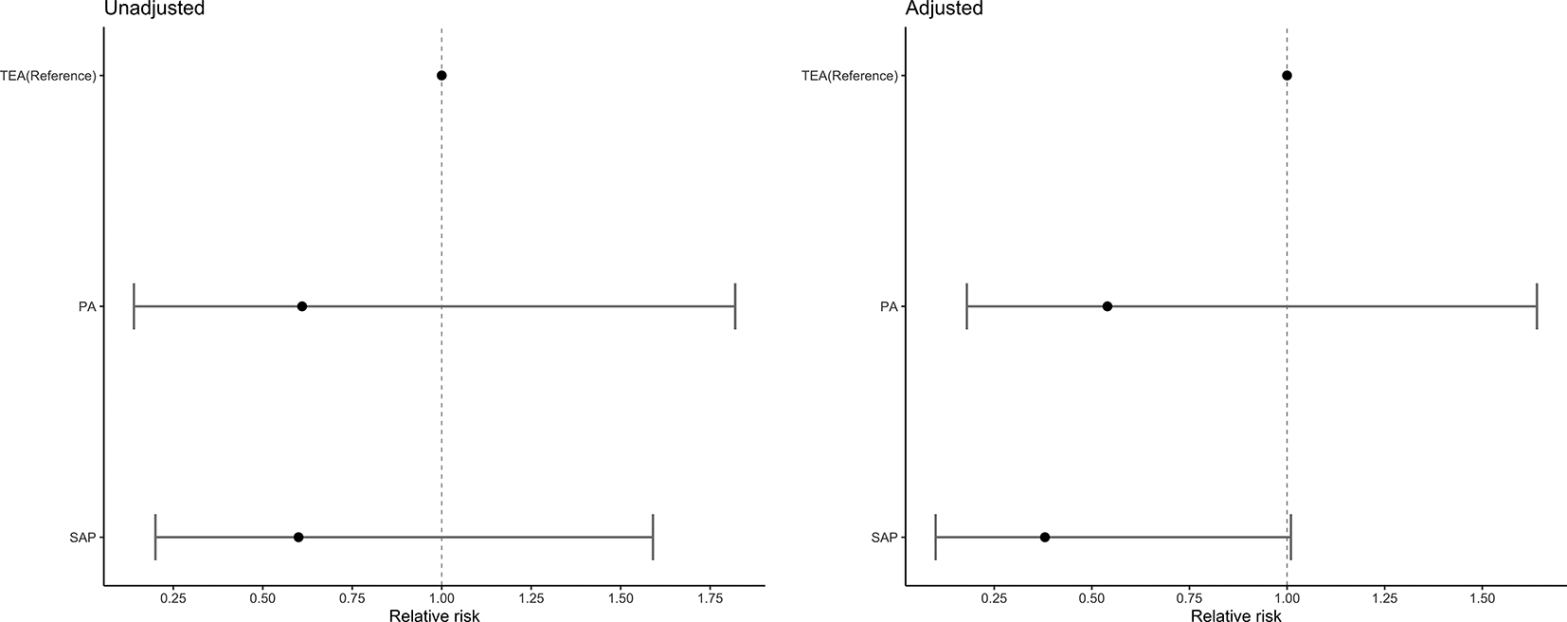
Relative risks (CIs) for the unadjusted and adjusted log binomial models comparing in-hospital mortality risk. Adjusted covariates: age, gender, ISS, RFS score, CCI, most severely injured body region (head, chest and other), surgical rib fixation and isolated chest injury versus polytrauma. CCI, Charlson Comorbidity Index; ISS, injury severity score; RFS, rib fracture score.

The overall mortality rate was 13.2%% (95% CI 7.8% to 18.7%), 4.59% (n=5) of participants died in the SAP group, 4.55% (n=3) in the PA group and 7.56% (n=12) in the TEA group. In the adjusted model, there were no differences in mortality across block types (figure 4).

### LOS in-hospital and critical care

**Figure 5:**
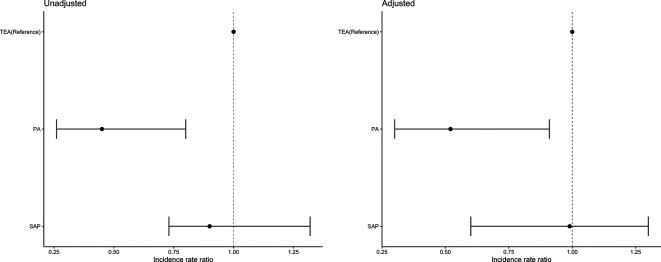
Incidence rate ratio (CIs) for the unadjusted and adjusted negative binomial model comparing length of stay hospital. Adjusted covariates: age, gender, ISS, RFS score, CCI, most severely injured body region (head, chest and other), surgical rib fixation and isolated chest injury versus polytrauma. CCI, Charlson Comorbidity Index; ISS, injury severity score; RFS, rib fracture score.

**Figure 6:**
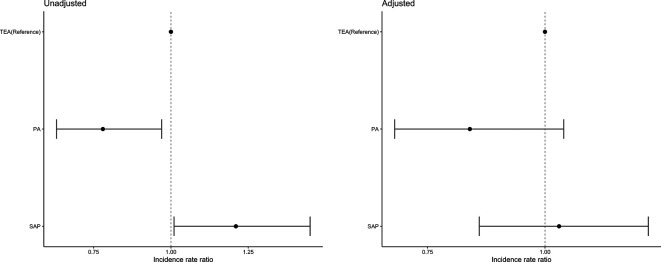
Incidence rate ratio (CIs) for the unadjusted and adjusted negative binomial model comparing length of stay in critical care. Adjusted covariates: age, gender, ISS, RFS score, CCI, most severely injured body region (head, chest and other), surgical rib fixation and isolated chest injury versus polytrauma. CCI, Charlson Comorbidity Index; ISS, injury severity score; RFS, rib fracture score.

The overall LOS in critical care mean (SD) was 7.8 days (7.5) and in-hospital LOS was mean (SD) 18.2 days (17.4). The mean (SD) LOS in critical care was highest in the TEA group (9.6 days (9.3)), followed by SAP (9.4 days (10.0)) and lowest in the PA group (4.3 days (3.2)). The mean (SD) hospital LOS was highest in the SAP group (22.1 days (16.9)), followed by TEA (18.3 days (15.6)) and lowest in the PA (14.3 days (19.7)). In an adjusted model, no significant differences were found between groups with respect to hospital LOS (figure 5). The PA group compared with TEA had a significantly shorter critical care stay (figure 6).

The complete results of the adjusted and unadjusted models with associated p values are available in the supplementary material for inspiratory volumes and pain scores (online supplementary table 1), mortality (online supplementary table 2) and LOS in critical or hospital (online supplementary table 3).

10.1136/rapm-2019-101119.supp1 Supplementary data

## Discussion

Our study suggests that all three techniques of regional anesthesia contributed to reductions in pain and improvements in respiratory function and that postinsertion SAP is likely to be comparable with TEA or PA when used for the analgesia of MRFs. This is the first pragmatic study that has assessed analgesic adequacy in those receiving SAP, TEA or PA and so adds to the growing body of literature that has detailed a significant improvement in pain scores and inspiratory volumes following SAP analgesia.^11 20 21^

Previous systematic reviews comparing traditional analgesic modalities have not reported significant differences in mortality or LOS.^12 13^ Our findings of a reduced rate of critical care LOS in the PA group should be interpreted with caution given the possibility of unmeasured confounding, lack of adjustment for multiple comparisons and secondary outcome metric status.

From a population health standpoint, our SAP block findings are encouraging given the current underutilization of traditional techniques (ie, TEA or PA) in the setting of trauma. For example, it has been reported that fewer than 3% of patients in England and Wales in 2017 with rib fractures received TEA or PA.^22^ This is likely due to contraindications, associated side effects such as hypotension and the advanced level of skill required for the insertion of PA and TEA analgesia. The fascial plane techniques (ie, SAP/ESP block), in comparison, are presumed to be safe and technically easy to insert given the superficial location and availability of ultrasound guidance. Although not examined in our study, SAP and ESP blocks have also been shown to reduce opioid consumption, which would be anticipated given the analgesic benefit demonstrated herein.^23 24^ SAP analgesia should not produce sympathetic block which accounts for its anticipated hemodynamic stability. Finally, given the superficial nature of the block, liberalization of anticoagulation rules for placement may be in order.

Our study has significant limitations. The heterogeneous nature of this cohort means that recommending a gold standard of analgesia is not possible.^25 26^ Given the observational nature of the data and inherent limitations of retrospective reviews, our data should be interpreted with caution due to the possibility of unmeasured confounders, multiple comparisons performed and small sample size for rare outcomes such as death. With respect to the heterogeneity of the data, the decision on the type of regional catheter used was left to the treating clinician.^27^ Additionally, we did not track or adjust for differences in bolus and infusion rates by block type.

Our study did not make comparison with a non-block group. Therefore, we are unable to comment on whether a block relative to pharmacological methods such as opioids adds value or improves outcomes. Despite these limitations, our study has several strengths including data collection across multiple sites and the paired nature of the data with respect to analgesic assessment and changes in respiratory mechanics.

### Conclusion

In two large tertiary care hospitals in the UK, regional anesthesia appears to result in improvements in analgesia and respiratory mechanics in patients suffering from traumatic rib fractures. Given inherent limitations of TEA and PA blocks, SAP blocks may represent an attractive analgesic option for these patients.
