# Supplementary material for: Multicenter longitudinal cross-sectional study comparing effectiveness of serratus anterior plane, paravertebral and thoracic epidural for the analgesia of multiple rib fractures
Source: Reg Anesth Pain Med. 2020 Mar 11;45(5):351–6. doi: 10.1136/rapm-2019-101119 (PMC8408582; doi:10.1136/rapm-2019-101119)
Supplement: Supplementary data [file rapm-2019-101119supp001.pdf]

Supplementary Table 1: Adjusted and unadjusted models comparing change scores in inspiratory volume and the number of participants reporting a change in their pain scores from severe/moderate to mild/none

	Unadjusted			Adjusted		
Inspiratory volume	Mean difference [a]	95%CI	P Value	Mean difference [a]	95%CI	P Value
TEA	Reference			Reference		
PA	35.4	-176.3 to 247.1	0.74	-3.3	-200.4 to 193.8	0.97
SAP	-34.4	-241.0 to 172.1	0.75	-72.7	-274.5 to 129.2	0.48
Male				Reference		
Female				-396.2	-578.3 to -214.1	<0.001
ISS score				-2.6	-13.7 to 8.5	0.65
RFS score				-7.1	-17.8 to 3.6	0.20
Age				-7.5	-12.43 to -2.65	0.003
Pain scores	RR [b]	95%CI	P Value	RR [b]	95%CI	P Value
TEA	Reference			Reference		
PA	0.9	0.6 to 1.3	0.58	0.8	0.5 to 1.1	0.17
SAP	0.7	0.4 to 1.1	0.09	0.8	0.4 to 1.1	0.12
Male				Reference		
Female				1.2	0.9 to 1.5	0.25
ISS score				1.0	1.0 to 1.0	0.07
RFS score				1.0	1.9 to 1.0	0.83
Age				1.0	1.0 to 1.0	0.01

Abbreviations: RR=relative risk; CI=confidence interval; ISS = Injury Severity Score; PA = Paravertebral; RFS = Rib Fracture Score; SAP = Serratus Anterior Plane; TEA = Thoracic Epidural

Alpha set to 0.05

a: Mean difference (B coefficient) calculated from linear regression models - Mean difference reflects the difference in means between groups

b: RR (exp(B) coefficient) calculated from log binomial models - RR reflects the difference in risk between groups.

Adjusted covariates: age, gender, ISS and RFS score.

Supplementary Table 2: Adjusted and unadjusted models comparing in-hospital Mortality risk

	Unadjusted			Adjusted		
	**RR**	**95%CI**	**P**	**RR**	**95%CI**	**P**
TEA	Reference			Reference		
PA	0.6	0.1 to 1.8	0.42	0.5	0.2 to 1.6	0.28
SAP	0.6	0.2 to 1.6	0.34	0.4	0.1 to 1.0	0.05
Gender – Male				Reference		
Gender – Female				0.4	0.2 to 1.1	0.07
ISS score				1.0	1.0 to 1.1	0.55
RFS score				1.0	0.9 to 1.0	0.49
Age				1.1	1.0 to 1.1	<0.01
CCI				1.3	1.0 to 1.5	0.02
Injured region– chest				Reference		
Injured region – head				0.4	<0.1 to 3.8	0.43
Injured region – other				0.2	<0.1 to 1.5	0.11
Surgical rib fixation – no				Reference		
Surgical rib fixation – yes				0.6	0.1 to 2.9	0.55
Isolated				Reference		
Poly trauma				1.7	0.6 to 4.6	0.29

Abbreviations: RR=relative risk; CI=confidence interval; CCI = Charlson Comorbidity Index; ISS = Injury Severity Score; PA = Paravertebral; RFS = Rib Fracture Score; SAP = Serratus Anterior Plane; TEA = Thoracic Epidural

Alpha set to 0.05

a: RR (exp(B) coefficient) calculated from log binomial models - RR reflects the difference in risk between groups.

Adjusted covariates: age, gender, ISS, RFS score, CCI, most severely injured body region (head, chest, other), surgical rib fixation and isolated chest injury versus polytrauma

Supplementary Table 3: Adjusted and unadjusted models comparing length of stay in critical care and hospital

	**Length of stay in critical care**						**Length of stay in hospital**					
	**Unadjusted**			**Adjusted**			**Unadjusted**			**Adjusted**		
	**IRR**	**95%CI**	**P-value** [a]	**IRR**	**95%CI**	**P-value** [a]	**IRR**	**95%CI**	**P-value** [a]	**IRR**	**95%CI**	**P-value** [a]
TEA	Reference			Reference			Reference			Reference		
PA	0.5	0.3 to 0.8	0.01	0.5	0.3 to 0.9	0.07	0.8	0.6 to 1.0	0.03	0.8	0.7 to 1.0	0.10
SAP	0.9	0.7 to 1.3	0.88	1.0	0.6 to 1.3	0.95	1.2	1.0 to 1.5	0.04	1.0	0.9 to 1.2	0.77
Gender – Male				Reference						Reference		
Gender – Female				0.9	0.7 to 1.3	0.73				1.6	1.1 to 1.6	<0.01
ISS score				1.0	1.0 to 1.0	0.98				1.0	1.0 to 1.0	0.07
RFS score				1.0	1.0 to 1.0	0.01				1.0	1.0 to 1.0	<0.01
Age				1.0	1.0 to 1.0	0.90				1.0	1.0 to 1.0	0.64
CCI				1.2	1.1 to 1.4	<0.001				1.2	1.1 to 1.2	<0.001
Injured region– chest				Reference						Reference		
Injured region – head				1.5	0.9 to 2.6	0.09				1.8	1.3 to 2.5	<0.01
Injured region – other				0.8	0.53 to 1.2	0.21				1.2	0.9 to 1.6	0.08
Surgical rib fixation – no				Reference						Reference		
Surgical rib fixation – yes				1.4	1.0 to 1.9	0.03				1.3	1.1 to 1.6	<0.01
Isolated				Reference						Reference		
Poly trauma				1.5	1.0 to 2.3	0.07				1.1	0.9 to 1.4	0.25

Abbreviations: IRR=Incidence Rate Ratio; CI=confidence interval; CCI = Charlson Comorbidity Index; ISS = Injury Severity Score; PA = Paravertebral; RFS = Rib Fracture Score; SAP = Serratus Anterior Plane; TEA = Thoracic Epidural

Alpha set to 0.05

a: IRR (exp(B) coefficient) calculated from negative binomial models - IRR reflects the difference in incidence of events between groups.

Adjusted covariates: age, gender, ISS, RFS score, CCI, most severely injured body region (head, chest, other), surgical rib fixation and isolated chest injury versus polytrauma.
